# Concurrent therapeutic and behavioral interventions are associated with a reduced number of emerging *Dracunculus medinensis* worms in dogs in Chad

**DOI:** 10.1371/journal.pntd.0012896

**Published:** 2026-02-02

**Authors:** Amy C. Dupper, Christopher A. Cleveland, Ellen K. Haynes, Michael J. Yabsley, Fernando Torres-Velez, Métinou Koumétio Sidouin, Philip Tchindebet Oaukou, Adam J. Weiss, Rebecca Garabed

**Affiliations:** 1 The Ohio State University, Columbus, Ohio, United States of America; 2 Southeastern Cooperative Wildlife Diseases Study, University of Georgia College of Veterinary Medicine, Athens, Georgia, United States of America; 3 Center for Ecology of Infectious Diseases, University of Georgia, Athens, Georgia, United States of America; 4 Warnell School of Forestry and Natural Resources, University of Georgia, Athens, Georgia, United States of America; 5 The Carter Center, Atlanta, Georgia, United States of America; 6 The Carter Center, N’Djamena, Tchad; 7 Programme National d’Éradication du Ver de Guinée, Ministère de la Santé Publique, et de la Prévention, N’Djamena, Tchad; Swiss Tropical and Public Health Institute: Schweizerisches Tropen- und Public Health-Institut, SWITZERLAND

## Abstract

*Dracunculus medinensis* (Guinea worm; GW) is a parasitic nematode that causes dracunculiasis (Guinea worm disease; GWD). The annual incidence of GWD in humans has been reduced by over 99.9% globally since the 1980s thanks to the implementation of complementary interventions. Dogs are now the primary hosts of GW and impede eradication efforts. The antihelmenthic drug, flubendazole (FLBZ), was suggested as a possible therapeutic intervention after it was found to be partially effective at reducing fertility of *D. medinensis* in experimentally infected ferrets. A 2019 clinical trial of FLBZ in Chad found no statistically significant difference in GW infections between treated and control dogs, but longer term effects may be observed if FLBZ reduced fertility of *D. medinensis.* This study leveraged surveillance data from the National Guinea Worm Eradication Program of the monthly count of *D. medinensis* worms in dogs between January 1, 2019, and September 30, 2021, for 56 villages to examine whether FLBZ would have an observable effect over 33 months and in the presence of another intervention, proactive tethering. We fit hypothesis-informed models of the combined interventions using negative binomial generalized linear mixed models. We averaged the top models together and predicted the number of *D. medinensis* infections per month for an average village. Based on the model predictions, we observed a clear delineation of effects between March and August 2021, approximately one year after most villages initiated proactive tethering and approximately two years after a few villages initiated FLBZ treatment. During this period, the predicted number of dog infections were reduced by 83% (95% CI, 76% to 88%) when using FLBZ and proactive tethering concurrently, by 63% when using FLBZ alone (95% CI: 44% to 75%), and by 55% when using proactive tethering alone (95% CI: 52% to 58%) compared to baseline control methods. When used together, proactive tethering and FLBZ may be important tools in reducing the village-level *D. medinensis* burden in dogs.

## Author summary

Guinea worm disease is caused by the nematode *Dracunculus medinensis* and is classically transmitted to the host by drinking water containing copepods infected with third-stage infectious *D. medinensis* larvae. Guinea worm disease in humans has been reduced by over 99% since it was targeted for eradication, but increased Guinea worm infections in dogs pose a challenge to the eradication of the disease. Flubendazole is a therapeutic intervention that was found to inhibit embryogenesis of *D. medinensis* in a ferret model, but a previous clinical trial of flubendazole in dogs found no difference in emerging *D. medinensis* worms between treated and control dogs. We sought to examine the effects of flubendazole alone and in the presence of another intervention, proactive tethering, by using village level data instead of dog level data. We found that flubendazole alone and when used along with proactive tethering resulted in the lowest number of *D. medinensis* in dogs beginning 22 months after the first injection of flubendazole. Understanding the individual and concurrent effects of intervention strategies may better inform eradication efforts.

## Introduction

Dracunculiasis (Guinea worm disease; GWD) is the disease caused by the parasitic nematode *Dracunculus medinensis.* The life cycle of *D. medinensis* is complex and requires an intermediate host [1,2]. An uncontained, gravid *D. medinensis* female can release anywhere from 500,000–3 million first-stage larvae (L1) into a body of water [3–5]. L1s can survive for up to one week in water but are capable of infecting copepods (order Cyclopoida), the intermediate host, for no more than five days at 24°C [4–6]. The larvae undergo two molts within the copepod to become third-stage infectious larvae (L3) [4]. At this point in the lifecycle, *D. medinensis* can be transmitted to human and animal hosts through the consumption of water containing copepods with L3s [4,5,7–11]. Between three and four months post ingestion of copepods with L3s, *D. medinensis* sexually mature and reproduce [4,5,8]. Around month 10, the embryos within the gravid female have developed into L1s, and the gravid female worm begins to move through the host’s subcutaneous tissues toward the site of emergence, most often the distal extremities [4,5,8]. Prior to emergence, a painful blister forms as a host response to a few L1s being released into the subdermis [8,12]. Exposing the blister to water causes the blister to rupture, and the gravid female worm expels the L1s into the water perpetuating the cycle [4,13]. The average prepatent period, or the time from ingestion of infected copepods to when L1s are expelled from the gravid female worm, is 12 months at the population level, but individuals can experience prepatent periods between 10–14 months [4,8].

GWD was targeted for eradication in 1980 by the Centers for Disease Control and Prevention (CDC) [14]. Since 1986, the Carter Center has partnered with ministries of health in endemic countries to lead the Guinea Worm Eradication Program with support from the World Health Organization [14,15]. The annual number of GWD cases has decreased globally from 3.5 million in 1986 to15 human cases in 2024 [15,16]. The drastic reduction in human cases of GWD has been accomplished thanks to the implementation of complementary interventions such as: treating water bodies with temephos (an organophosphate larvicide), providing villages with safe drinking water, communicating behavior change (such as filtering drinking water), and case and infection management [17,18]. Despite the reduction in human cases, Angola, Chad, Cameroon, Mali, and South Sudan reported 600 dog and 66 cat infections in 2024 [19]. An alternative mode of transmission via consumption of an aquatic paratenic or transport host has been proposed to explain the increase in non-human animal GW infections despite low case numbers in humans [20–23], and GW infections in dogs are believed to be driving human cases of GWD [24,25].

Following a 10-year absence of GWD in humans, during which Chad transitioned from active to passive surveillance, ten *D. medinensis* worms in eight villages were confirmed emerging from humans in 2010 [26,27], and the first laboratory confirmed *D. medinensis* worm emerged from a Chadian dog in 2012 [24]. Beginning in February 2015, the National Guinea Worm Eradication Program of Chad (identified by its French acronym, PNEVG-T) gave monetary incentives to villagers to adopt additional interventions in an effort to reduce transmission of GWD in high-risk areas, including burying fish entrails and tethering dogs and cats with a confirmed GW infection [14,28]. In March 2020, PNEVG-T launched the proactive tethering program in villages with at least five dog GW infections per year, and by the end of 2023, 433 villages were proactively tethering dogs and cats [29,30]. Chad experienced the highest number of dog GW infections in 2019 with a total of 1,934 dogs detected with GW infections that year [29]. Since then, the annual number of dog GW infections has decreased, and, in 2024, Chad reported 234 dog infections and 47 peri-domestic cat infections [16].

Flubendazole (FLBZ) is a benzimidazole anthelmintic that effectively treats some filarial parasites in humans and non-human animals by interrupting embryogenesis and larval development [31,32]. A single subcutaneous injection into the fatty tissue, or depot site, enables the slow release of the drug over time allowing greater bioavailability for parasites residing outside the gut [33]. FLBZ was partially effective in reducing the fertility of *D. medinensis* in laboratory-infected ferrets [34]. These laboratory findings prompted a field study in Chad in 2019, which included 23 villages with a high burden of *D. medinensis* in dogs [34]. On enrollment in May 2019, half of the dogs in each village were given one subcutaneous injection of FLBZ (15 mg/kg) for three consecutive days, while the other half of dogs in the village were given a placebo [34]. This administration schedule was repeated twice more, at a follow-up visit in November 2019 and again at the final follow-up visit in June 2020 [34]. The field study followed enrolled dogs for 12 months (first-generation life cycle of *D. medinensis*) and targeted different stages of the developing *D. medinensis* within the host; however, they found no difference in the number of emerging *D. medinensis* worms between individual dogs receiving FLBZ and dogs receiving placebo [34].

The reduced fertility of *D. medinensis* larvae from gravid female worms extracted from FLBZ-treated ferrets indicated a reduced potential of transmission, which may be observed at the population level beyond 12 months after the start of treatment with FLBZ. Therefore, a second FLBZ trial was conducted between October 2021 and May 2023 where treatment assignment was randomized at the village level. The present study analyzed village level monthly surveillance data of villages enrolled in the second FLBZ trial between January 2019 and September 2021, before the start of the second trial. This study used observational data, and we, therefore, were unable to account for all interventions implemented at the time of the study that may also have contributed to decreases in emerging *D. medinensis* worms in dogs. Our study aimed to estimate: 1) the post-treatment timing of when FLBZ is thought to act on *D. medinensis* within the host, 2) the post-intervention timing of when proactive tethering has the greatest effect on emerging *D. medinensis* worms, and 3) concurrent population effects of FLBZ and tethering in reducing emerging *D. medinensis* worms in dogs based on a predictive model.

## Methods

### Ethics statement

The proactive tethering program is run and implemented by the PNEVG-T outside of this study. The welfare of tethered animals is monitored by the PNEVG-T veterinarian who can be contacted by village volunteers if there is a concern. While no formal study has been done, observationally, dogs have reduced incidents of many infectious diseases, trauma from vehicles, and interactions with wild predators and snakes. Concerns on increased internal parasite load have not been confirmed in a small sample of tethered animals that have been tested or in broader testing, which is ongoing. Furthermore, PNEVG-T has implemented deworming and other health initiatives to support the welfare of these dogs.

The procedures described as part of the 2019 FLBZ trial in dogs and ferrets were reviewed and approved by the University of Georgia’s institutional animal care and use committee (A2019 04–005-Y3-A0 and A2020 05–003-Y1-A0). Data for timing of proactive tethering were collected with approval from the National Bioethics Committee of the Chadian Ministry of Higher Education, Research and Innovation (005/PR/MESRI/SE/DGM/CNBT/SG/2022). No interventions were part of this secondary analysis of observational data collected by the program for programmatic purposes.

### Study population

**Fig 1:**
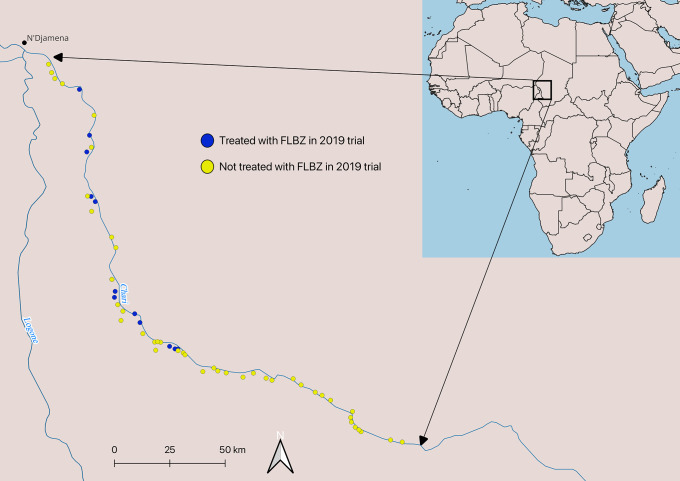
Map of village locations in the study area. The blue line represents the Chari River. All villages are clustered along the west side of the Chari River. Made with Natural Earth. Free vector and raster map data @ naturalearthdata.com.

Outcome data on the monthly count of dog GW infections per village were obtained from the PNEVG-T. These data are part of regular monthly active surveillance data collected by PNEVG-T. Villages where GWD is endemic and transmission risk is high are under active surveillance [35]. In villages under active surveillance, volunteers search house-to-house for GWD and signs thereof 3–4 times per week and reported all instances of emerging worms in humans, dogs, and cats [35]. PNEVG-T field supervisors each supervise village volunteers in 8–15 villages, and all emerging *D. medinensis* reports were shared with regional PNEVG-T offices, where quality checks were performed [35,36]. Data used for this study included PNEVG-T’s monthly village level counts of emerging *D. medinensis* worms but did not include dog-level data. The villages selected for inclusion in the present study (N = 56) were those randomly selected for inclusion in the 2021 field trial, of which 12 villages (21.4%) were enrolled in the 2019 FLBZ field trial and 44 villages (78.6%) were not enrolled in the 2019 field trial (Fig 1). Their inclusion in the 2021 field trial was based on their being located in a highly endemic section of the west bank of the Chari River south of N’Djamena, Chad and north of Bousso that was accessible throughout the year, considered to have consistent surveillance intensity, and are assumed to be similar beyond the interventions.

### Data

**Fig 2:**
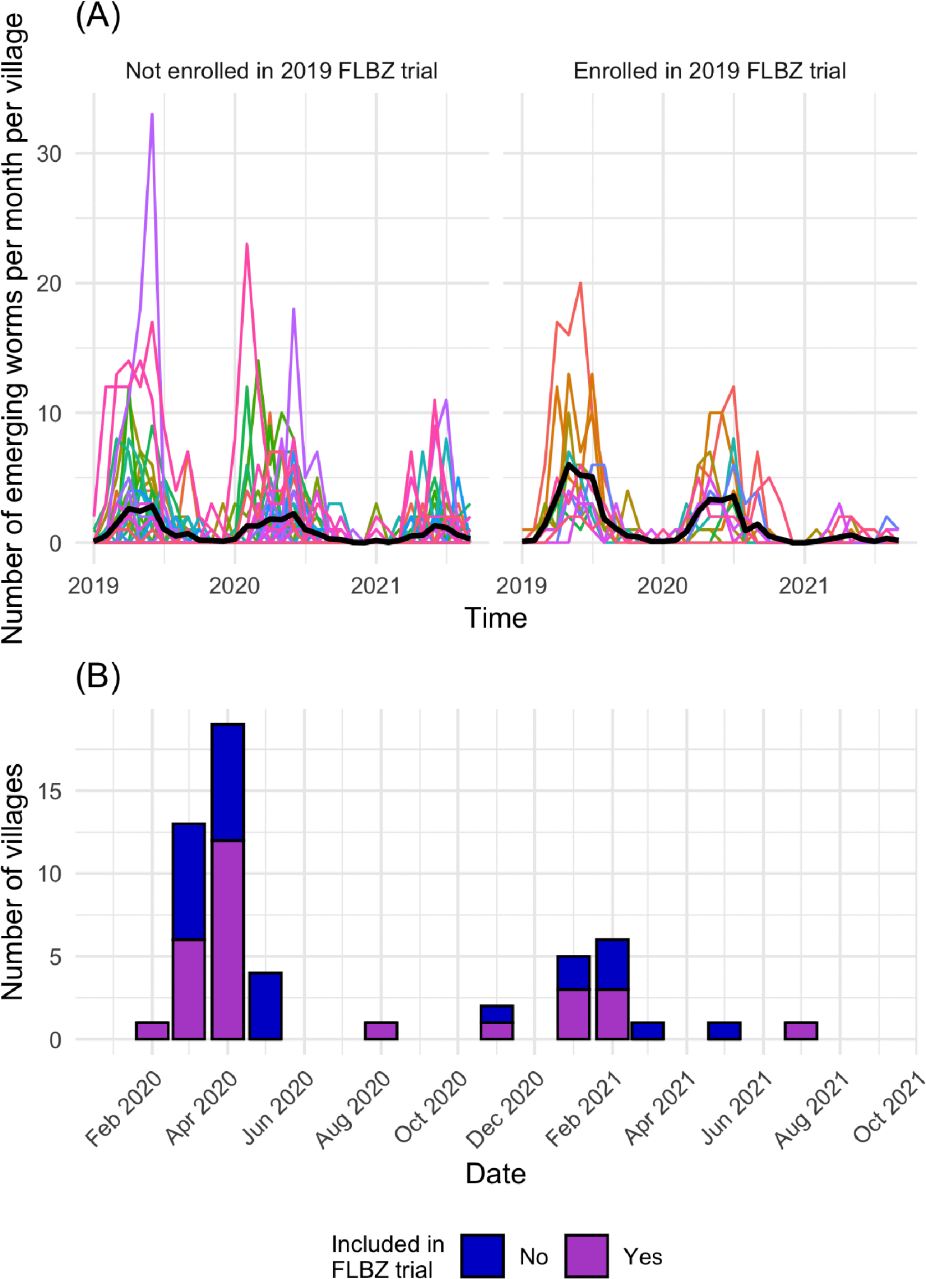
Descriptives of villages included in the study analysis. **(A)** Spaghetti plots of the monthly count of emerging *D. medinensis* in dogs in each village by enrollment status in the 2019 trial. The black line represents the mean number of worms per month over all villages. **(B)** Bar graph of the number and month villages began proactively tethering dogs by enrollment status in the 2019 FLBZ trial.

The main outcome of interest was the monthly number of emerging *D. medinensis* worms from dogs in the 56 villages. The primary exposures of interest were FLBZ treatment and proactive tethering at the village level. Half of dogs in each village in the FLBZ 2019 field trial were randomized to treatment with FLBZ, so villages where any dogs were treated with FLBZ were considered to be FLBZ-treated villages in this study (Fig 2A). Because the study design of the 2019 field trial randomized individual dogs in each village to receive either FLBZ or a placebo, teasing out the effects of FLBZ from the effects of the trial when using monthly data at the village level will remain a limitation of this study.

The data on timing of proactive tethering were collected in the field under a research permit (005/PR/MESRI/SE/DGM/CNBT/SG/2022). All but two villages in this study initiated proactive tethering between February 2020 and July 2021 (Fig 2B). The month and year of when each village initiated proactive tethering of dogs at the village level was obtained by asking village supervisors and individual dog owners. If the reported month and year when proactive tethering was initiated differed among dog owners, the month most frequently reported was selected as the village start date.

### FLBZ mechanism of action hypotheses

**Fig 3:**
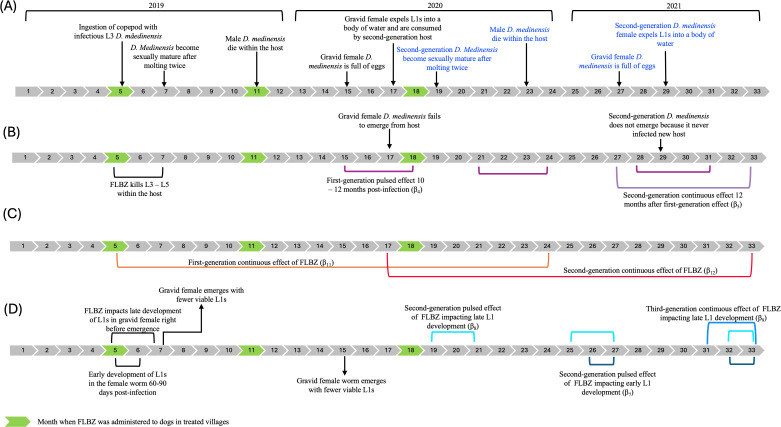
Graphic of the different hypotheses for when FLBZ may have an effect. **(A)** Hypothesized timeline of the first-generation (black text) and second-generation (blue text) life cycle of *D. medinensis*. **(B)** Timeline showing the hypothesized first- and second-generation effects of FLBZ if it kills developing L3-L5 *D. medinensis* within the host. **(C)** Timeline showing the hypothesized first- and second-generation effects of FLBZ if it had a continuous effect over six months on the developing *D. medinensis* within the host. **(D)** (Bottom) Timeline of the hypothesized second-generation effects of FLBZ if it killed developing L1s in the gravid female. **(D)** (Top) Timeline of the hypothesized second- and third-generation effects of FLBZ if it killed developing L1s in the gravid female worm right before emergence from the host.

The exact mechanism of how FLBZ acts on *D. medinensis* within the host remains unknown, so we solicited the opinion of seven members of the Guinea Worm Therapeutics Working Group. This group posited three separate hypotheses for how FLBZ could affect the developing *D. medinensis* within the host: 1) FLBZ kills developing L3 – L5 within the host, 2) FLBZ has a continuous effect over six months on the developing *D. medinensis*, or 3) FLBZ kills developing L1s in the gravid female*.* A graphic of the different proposed mechanisms is shown in Fig 3. While several second- and third-generation effects were expected to extend beyond September 2021, we chose to end our analysis at this time point.

1. FLBZ kills L3 – L5 within the host

Under this hypothesis, FLBZ is assumed to remain in the host tissues for at least one month after injection and is thought to interrupt transmission by killing the developing *D. medinensis* L3 – L5 within the host (Fig 3B). If a dog is either recently infected with *D. medinensis* or is infected with *D. medinensis* shortly after FLBZ injection, when it is between the L3 and L5 stage, we assume that *D. medinensis* will be killed within the host, never reproduce, and never emerge. We would expect to observe a reduction in the number of emerging *D. medinensis* beginning 10 months after injection. Because these worms never emerge, the life cycle is interrupted, and a second-generation effect should be observed beginning March 2021, 12 months after the first-generation effect.

2. FLBZ has a continuous effect over six months

The FLBZ injection administered to dogs in the 2019 trial was formulated to release slowly into the hosts’ tissues over six months from a depot site [34]. Under this hypothesis, FLBZ would exhibit an immediate and continuous effect on the developing *D. medinensis* within the host over 18 months (the last injection was administered in June 2020), regardless of the *D. medinensis* life stage. The FLBZ injections were administered every six months, so we would expect to see a reduction in the number of emerging *D. medinensis* worms beginning May 2019 (Fig 3C). Assuming a reduction of *D. medinensis* in the environment, we would expect to observe an additional second-generation effect of FLBZ beginning May 2020, 12 months after the first injection.

3. FLBZ impacts L1 development in gravid female

Under this hypothesis, FLBZ is thought to impact the developing larvae (L1) within the gravid female *D. medinensis*, whereby the L1s would have reduced viability. Assuming FLBZ remains in the dogs’ tissues for at least one month, FLBZ could either impact early or late development of L1s. If FLBZ impacts early development of L1s in the gravid female worm, the female worm would be exposed to FLBZ shortly after reproducing when L1s begin to develop (Fig 3D, bottom). Approximately nine months after treatment, the gravid *D. medinensis* female would emerge, expelling less viable L1s into the water. These L1s would fail to infect a copepod, so we expect to see a second-generation effect beginning 21 months after the first injection of FLBZ. Our analysis time does not allow us to observe a third-generation effect for this hypothesis. Similarly, if FLBZ impacts later L1 development right before the female worm emerges, 10–12 months post-infection, a second-generation effect would be observed beginning 14 months after the first FLBZ injection (Fig 3D, top). As L1s have reduced viability, transmission would be reduced such that an additional third-generation effect would be observed beginning July 2021.

### Proactive tethering hypotheses

Villages began proactive tethering dogs at different times, and only two villages had not officially adopted proactive tethering during the study period (Fig 2B). We hypothesized, first, that within the first 12 months after the start of proactive tethering, the number of reported worms emerging from dogs would increase relative to the prior year because any emerging *D. medinensis* worms would be more easily observed in dogs due to them being tethered. Given the prepatent period of *D. medinensis,* we also hypothesized that the reported number of worms emerging from dogs would decrease beginning 12 months after initiating proactive tethering (second-generation), because dogs would be prevented from accessing uncontrolled sources of infection, including shared bodies of water. Candidate models for proactive tethering included a combination of these hypotheses (Table 1).

**Table 1:** List of fixed effects included in the candidate models.

Hypotheses	Coefficients for fixed effects	Definition
Variables included in the base model	$\beta_{\text{1}}$	Inclusion in 2019 FLBZ trial (binary variable; 1 if yes vs 0 if no)
	$\beta_{\text{2}}$	Seasonal effect estimated via decomposition
	$\beta_{\text{3}}$	Study timestep (in month) (continuous, January 2019 = 1 and September 2021 = 33)
FLBZ kills L3 – L5 within the host	$\beta_{\text{4}}$	Pulsed first generation effect of FLBZ in treated villages (binary variable; 1 for March – June 2020, September – December 2020, and April – July 2020, and 0 otherwise)
	$\beta_{\text{5}}$	Second generation effect of FLBZ in treated villages (binary variable; 1 for March 2021 – September 2021, and 0 otherwise)
	$\beta_{\text{6}}$	Time since second generation effect of FLBZ (continuous variable beginning March 2021 – September 2021, and 0 otherwise)
FLBZ affects early development of L1s in gravid female *D. medinensis*	$\beta_{\text{7}}$	Pulsed second generation effect of FLBZ in treated villages (binary variable; 1 for February – March 2021 and August – September 2021, and 0 otherwise)
FLBZ affects late development of L1s in gravid female *D. medinensis*	$\beta_{\text{8}}$	Pulsed second generation effect of FLBZ in treated villages (binary variable; 1 for July – September 2020, January – March 2021, and August – September 2021, and 0 otherwise)
	$\beta_{\text{9}}$	Third generation effect of FLBZ in treated villages (binary variable; 1 for July – September 2021, and 0 otherwise)
	$\beta_{\text{1}\text{0}}$	Time since third generation effect of FLBZ in treated villages (continuous variable beginning July – September 2021, and 0 otherwise)
FLBZ has a continuous 6-month effect on developing *D. medinensis*	$\beta_{\text{1}\text{1}}$	Continuous first-generation effect of FLBZ in treated villages (binary variable; 1 for May 2019 – December 2020, and 0 otherwise)
	$\beta_{\text{1}\text{2}}$	Continuous second-generation effect of FLBZ in treated villages (binary variable; 1 for May 2020 – September 2021, and 0 otherwise)
	$\beta_{\text{1}\text{3}}$	Time since second generation effect of FLBZ in treated villages (continuous variable beginning May 2020 – September 2021, and 0 otherwise)
Proactive tethering has an immediate effect on emerging *D. medinensis*	$\beta_{\text{1}\text{4}}$	First-generation effect of proactive tethering (binary variable; 1 beginning the month each village initiated proactive tethering, and 0 otherwise)
Proactive tethering has a delayed effect on emerging *D. medinensis*	$\beta_{\text{1}\text{5}}$	Second-generation effect of proactive tethering (binary variable; 1 beginning 12 months after village initiated proactive tethering, and 0 otherwise)
Proactive tethering has a continuous delayed effect on emerging *D. medinensis*	$\beta_{\text{1}\text{6}}$	Time since second-generation effect of proactive tethering (continuous variable; 1 beginning 12 months after village initiated proactive tethering, and 0 otherwise)
	$b_{\text{0}i}$	Random effect for village

### Statistical analyses

Analyses and data visualizations were performed in R (version 4.3.2), and Fig 1 was made in QGIS (version 3.38.1-Grenoble) using Natural Earth Data. Because GWD exhibits a strong seasonal trend, we generated a time series of the monthly number of emerging *D. medinensis* for all villages in the study, and we then estimated the seasonal trend using the ‘decompose’ function in the *stats* package [37]. All models were assessed for collinearity using the ‘check_collinearity’ function in the *performance* package [38], and variables with a variance inflation factor (VIF) greater than 10 were determined to be multicollinear [39]. The interactions with time tended to cause multicollinearity issues, so these variables were re-coded to address these issues (i.e., time since second generation effect of proactive tethering) after which the variables were no longer highly correlated. Additionally, models were first run as Poisson models and then as negative binomial models and compared using a likelihood ratio test. In all instances, negative binomial models performed better than Poisson models (S1 Fig). We elected not to include the number of dogs in each village as an offset because the village dog census does not accurately reflect the true dog population in each village. This is because village supervisors who collect these data have different methods for counting dogs, and including dog population estimates would introduce more bias into the model. We instead included a village level random effect to capture the variability in dog populations.

We identified 28 candidate models that represented all possible FLBZ and proactive tethering hypotheses using negative binomial generalized linear mixed models (GLMMs). All models included fixed effects for study timestep (in month, continuous), a seasonal effect estimated via decomposition, and a random effect for village. Each model included a combination of fixed effects for FLBZ and proactive tethering based on hypotheses when each intervention is thought to have an effect on emerging *D. medinensis* (Table 1)*.* All model predictions that included proactive tethering used April 2020 as the start date for all villages because this was the mode of when villages began proactively tethering dogs. We screened these 28 combined models to select the best fitting models by comparing the difference in AICc and the AICcw_i_. We selected the models with a ΔAICc < 7 that also added up to an AICcw_i_ of at least 95% [40]. The weights of each of the individual predictors after model averaging were examined, and predictors with AICcw_i_ ≤ 0.02 were dropped from the model averaged predictions. We then calculated the predicted number of emerging *D. medinensis* worms per month for an average village without FLBZ or proactive tethering, using FLBZ only, using proactive tethering only, and combining FLBZ treatment and proactive tethering using the ‘modavgPred’ function in the *AICcmodavg* package [41]. Predictions were calculated on the full model averages, where all models are assumed to include all variables, but some might be set to zero to reduce model selection bias. Standard errors of the weighted fixed effects were extracted and used to calculate confidence intervals on the predictions. Model covariates with a p-value less than 0.05 and model predictions with non-overlapping confidence intervals are considered significant.

## Results

### Demographics

This study included 56 villages, of which 12 villages (21.4%) were enrolled in the 2019 FLBZ field study and 44 villages (78.6%) were not enrolled in the 2019 field study. Between January 2019 and September 2021, FLBZ-treated villages had an median of 62 dogs per village (Interquartile range (IQR): 42 – 82), while control villages for FLBZ had an median of 49 dogs per village (IQR: 17 – 108). According to surveillance data, the total number of emerging *D. medinensis* from dogs in FLBZ-treated villages was 532, and dogs in control villages had a total of 1,242 emerging *D. medinensis*. Dogs in FLBZ-treated villages had an average of 1.3 (standard deviation [SD] = 2.7) emerging *D. medinensis* per month, while dogs in control villages had an average of 0.9 (SD = 2.3) emerging *D. medinensis* per month over the study period. All villages, other than two, enrolled in the proactive tethering program during the study period. Prior to proactive tethering, villages had an average of 1.1 (SD = 2.8) emerging *D. medinensis* worms per month, compared to an average of 1.7 (SD = 1.6) emerging *D. medinensis* worms per month after enrolling in the proactive tethering program.

### Impact of FLBZ

Of the candidate models that fit our selection criteria, only one model (Model 6) included fixed effects hypothesizing that FLBZ acts by killing the developing L3 – L5 *D. medinensis* female within the host, thus preventing *D. medinensis* from emerging (Table 2). Under this assumption, we assumed FLBZ would have an effect on the first-generation of emerging *D. medinensis* and an effect on the second-generation of emerging *D. medinensis*, decreasing the number of *D. medinensis* emerging over time. The remaining models all included fixed effects hypothesizing that FLBZ would have an immediate and continuous effect on *D. medinensis* within the host due to the slow release of FLBZ from a depot site over six months. Models 22 and 24 both only included a fixed effect assuming FLBZ would only affect the first-generation, or the first year, of emerging *D. medinensis*, while models 26, 27, and 28 included fixed effects hypothesizing FLBZ would affect the first-generation and second-generation, or the second year, of emerging *D. medinensis* and that this effect would continue over time.

**Table 2:** AICc, ΔAICc, and AICcw_i_ values of all candidate models.

Model #	Base Model	FLBZ effects	Proactive tethering effects	K	AICc	ΔAICc	AICcw_i_
26	b_1_ + b_2_ + b_3_	b_11_ + b_12_ + b_13_	b_14_ + b_15_	11	3983.09	0.00	0.50
28	b_1_ + b_2_ + b_3_	b_11_ + b_12_ + b_13_	b_14_ + b_15_ + b_16_	12	3984.72	1.62	0.22
27	b_1_ + b_2_ + b_3_	b_11_ + b_12_ + b_13_	b_15_ + b_16_	11	3985.39	2.30	0.16
22	b_1_ + b_2_ + b_3_	b_11_	b_14_ + b_15_	9	3988.21	5.12	0.04
6	b_1_ + b_2_ + b_3_	b_4_ + b_5_ + b_6_	b_14_ + b_15_	11	3989.55	6.45	0.02
24	b_1_ + b_2_ + b_3_	b_11_	b_14_ + b_15_ + b_16_	10	3989.98	6.88	0.02
23	b_1_ + b_2_ + b_3_	b_11_	b_15_ + b_16_	9	3990.14	7.05	0.01
25	b_1_ + b_2_ + b_3_	b_11_ + b_12_ + b_13_	b_14_	10	3990.34	7.25	0.01
8	b_1_ + b_2_ + b_3_	b_4_ + b_5_ + b_6_	b_14_ + b_15_ + b_16_	12	3991.03	7.94	0.01
7	b_1_ + b_2_ + b_3_	b_4_ + b_5_ + b_6_	b_15_ + b_16_	11	3991.85	8.76	0.01
18	b_1_ + b_2_ + b_3_	b_8_ + b_9_ + b_10_	b_14_ + b_15_	11	3995.04	11.94	0.00
10	b_1_ + b_2_ + b_3_	b_7_	b_14_ + b_15_	9	3995.19	12.10	0.00
20	b_1_ + b_2_ + b_3_	b_8_ + b_9_ + b_10_	b_14_ + b_15_ + b_16_	12	3996.25	13.16	0.00
12	b_1_ + b_2_ + b_3_	b_7_	b_14_ + b_15_ + b_16_	10	3996.70	13.61	0.00
21	b_1_ + b_2_ + b_3_	b_11_	b_14_	8	3996.92	13.82	0.00
19	b_1_ + b_2_ + b_3_	b_8_ + b_9_ + b_10_	b_15_ + b_16_	11	3996.94	13.85	0.00
11	b_1_ + b_2_ + b_3_	b_7_	b_15_ + b_16_	9	3997.09	13.99	0.00
5	b_1_ + b_2_ + b_3_	b_4_ + b_5_ + b_6_	b_14_	10	3997.30	14.21	0.00
14	b_1_ + b_2_ + b_3_	b_8_	b_14_ + b_15_	9	3997.44	14.35	0.00
2	b_1_ + b_2_ + b_3_	b_4_	b_14_ + b_15_	9	3997.73	14.64	0.00
16	b_1_ + b_2_ + b_3_	b_8_	b_14_ + b_15_ + b_16_	10	3999.26	16.16	0.00
4	b_1_ + b_2_ + b_3_	b_4_	b_14_ + b_15_ + b_16_	10	3999.49	16.39	0.00
15	b_1_ + b_2_ + b_3_	b_8_	b_15_ + b_16_	9	3999.62	16.52	0.00
3	b_1_ + b_2_ + b_3_	b_4_	b_15_ + b_16_	9	3999.80	16.71	0.00
17	b_1_ + b_2_ + b_3_	b_8_ + b_9_ + b_10_	b_14_	10	4006.04	22.94	0.00
9	b_1_ + b_2_ + b_3_	b_7_	b_14_	8	4008.59	25.50	0.00
1	b_1_ + b_2_ + b_3_	b_4_	b_14_	7	4009.85	26.76	0.00
13	b_1_ + b_2_ + b_3_	b_8_	b_14_	8	4011.03	27.94	0.00
Base	b_1_ + b_2_ + b_3_			5	4011.41	28.31	0.00

Abbreviations: AICc, Akaike Information Criterion corrected for small sample sizes; ΔAICc, difference in corrected Akaike Information Criterion; AICcw_I_, weights for the corrected Akaike Information Criterion; K, number of parameters in the model; FLBZ, flubendazole.

All models included a random effect for village.

The fixed effects hypothesizing that FLBZ kills the developing L3 – L5 *D. medinensis* within the host contributed very little weight (2%) to the model averaged fixed effects and were dropped when performing model predictions. Based on the model-averaged fixed effects, time since the second-generation effect of FLBZ in treated villages resulted in a significant decrease in the number of emerging *D. medinensis*, while season had a significant positive association with the number of emerging *D. medinensis* (Table 3). The predicted number of emerging *D. medinensis* females peaked in June 2020 when using just FLBZ compared to using just proactive tethering, using both proactive tethering and FLBZ, and using neither proactive tethering or FLBZ before decreasing in June 2021.

**Table 3:** Model averaged fixed effects for the count of emerging *D. medinensis* worms.

Variable	Ratio of Counts Estimate	p-value	Sum of Weights
Inclusion in 2019 FLBZ trial ($\beta_{\text{1}}$)	1.94	0.09	1.00
Seasonal effect ($\beta_{\text{2}}$)	2.29	<0.01	1.00
Study timestep (in month) ($\beta_{\text{3}}$)	0.98	0.07	1.00
Pulsed 1^st^-generation effect of FLBZ in treated villages ($\beta_{\text{4}}$)*	1.00	0.94	0.02
Pulsed 2^nd^-generation effect of FLBZ in treated villages ($\beta_{\text{5}}$)*	0.99	0.91	0.02
Time since 2^nd^-generation effect of FLBZ in treated villages ($\beta_{\text{6}}$)*	1.00	0.94	0.02
Continuous 1^st^-generation effect of FLBZ in treated villages ($\beta_{\text{1}\text{1}}$)	1.22	0.46	0.98
Continuous 2^nd^-generation effect of FLBZ in treated villages ($\beta_{\text{1}\text{2}}$)	1.37	0.31	0.92
Time since 2^nd^-generation effect of FLBZ in treated villages ($\beta_{\text{1}\text{3}}$)	0.91	0.03	0.92
1^st^-generation effect of proactive tethering ($\beta_{\text{1}\text{4}}$)	0.80	0.21	0.83
2^nd^-generation effect of proactive tethering ($\beta_{\text{1}\text{5}}$)	0.50	0.02	1.00
Time since 2^nd^-generation effect of proactive tethering ($\beta_{\text{1}\text{6}}$)	1.03	0.70	0.42

Abbreviations: FLBZ, flubendazole.

* These variables were not included in model-averaged predictions.

### Impact of proactive tethering

All candidate models also included a combination of fixed effects for when proactive tethering was thought to have an effect on *D. medinensis.* Models 6, 22, and 26 all include fixed effects hypothesizing an immediate effect of proactive tethering beginning the month when the village was enrolled in the proactive tethering program and a delayed effect of proactive tethering beginning 12 months after the month when the village was enrolled in the program. Models 24 and 28 had the same assumptions as models 6, 22, and 26 but with the addition of an interaction between the delayed effect and time. Model 27 included fixed effects assuming proactive tethering would only have an observed effect on emerging *D. medinensis* beginning 12 months after enrolling in the proactive tethering program and a decreasing effect over time. The variable for the second-generation effect of proactive tethering was significantly associated with a decrease in the number of emerging *D. medinensis* in the averaged model.

### Model predictions

**Fig 4:**
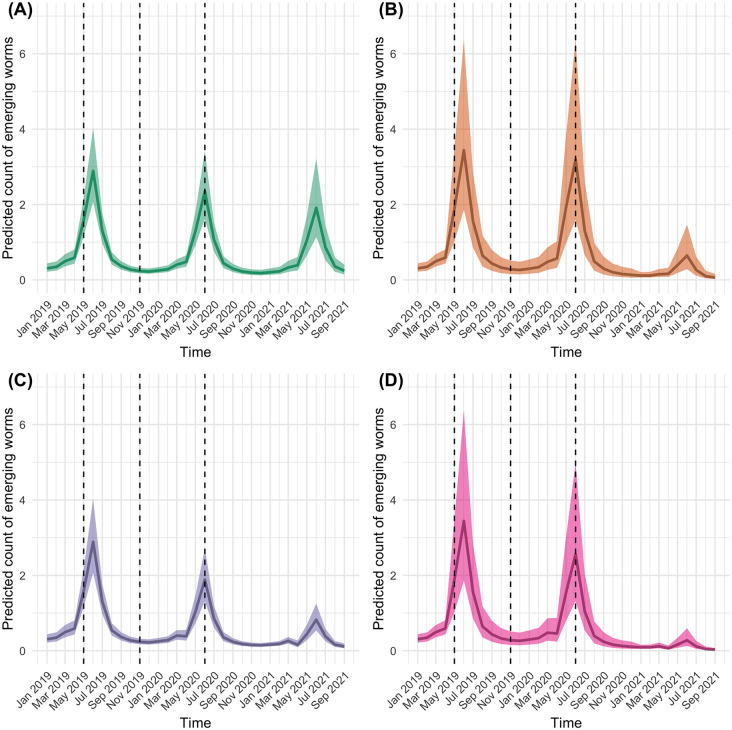
Predicted monthly count of emerging *D. medinensis* worms between January 2019 through September 2021. **(A)** Villages not using proactive tethering or FLBZ, **(B)** using only FLBZ, **(C)** using only proactive tethering, and **(D)** using both proactive tethering and FLBZ. The vertical dashed lines indicate months when FLBZ was administered to treated dogs. All predictions that include proactive tethering were set to initiate proactive tethering in April 2020.

Using the full model averaged fixed effects, the predicted number of emerging *D. medinensis* were not significantly different for any of the intervention scenarios over the first two seasonal peaks. It was not until the third peak between March 2021 and August 2021 that a clear difference in the predicted number of *D. medinensis* infections between intervention scenarios was seen. Concurrent use of proactive tethering and FLBZ resulted in the lowest predicted number of *D. medinensis* for the average village (83 emerging worms per 100 villages, 95% CI, 36 emerging worms to 195 emerging worms per 100 villages) compared to the absence of these interventions (488 emerging worms, 95% CI, 297 emerging worms to 801 emerging worms per 100 villages) followed by proactive tethering only (221 emerging worms, 95% CI, 144 emerging worms to 340 emerging worms per 100 villages) and FLBZ only (181 emerging worms, 95% CI, 74 emerging worms to 447 emerging worms per 100 villages) between March and August 2021 (Fig 4).

## Discussion

This study aimed to address outstanding questions about the timing and effectiveness of an existing behavioral intervention (proactive tethering) and a novel therapeutic intervention (FLBZ) on the predicted number of emerging *D. medinensis* in dogs when used separately and concurrently. The model-averaged fixed effects showed that when used concurrently, FLBZ and proactive tethering resulted in the lowest predicted number of emerging *D. medinensis* for the average village 22 months after the first treatment with FLBZ between March and August 2021, compared to each intervention used separately and when using neither of these interventions. The increase in the predicted number of emerging *D. medinensis* worms in June 2020 and the decrease in June 2021 under the FLBZ only scenario suggests that FLBZ has an effect on emerging *D. medinensis.* However, dog owners in villages involved in the 2019 FLBZ trial may have been more adherent in proactively tethering their dogs with study teams visiting every six months compared to villages not enrolled in the trial. Not being able to separate the effect of FLBZ from a possible effect due to increased programmatic presence in villages during the 2019 trial remains a limitation of this study. The villages included in this analysis were included in a second FLBZ clinical trial that began in October 2021 and ended in May 2023. The results from this trial are being analyzed as of the writing of this paper to further tease out the effects of FLBZ from the effects of FLBZ trial research activities at the population level.

The first clinical trial of FLBZ in dogs in Chad did not find a difference in the number of *D. medinensis* cases between FLBZ-treated dogs and control dogs over 12 months [34]. Therefore, this study sought to examine the second-generation effects of FLBZ using village level monthly surveillance data over 33 months. The predictions calculated from the model averaged fixed effects support the hypothesis that FLBZ and proactive tethering have an observed effect on the second-generation of emerging *D. medinensis*. Because the life cycle of *D. medinensis* takes approximately 10–14 months, an effect seen around 22 months post-treatment is consistent with the hypothesis that FLBZ inhibits embryogenesis and impairs motility of the immature larvae after expulsion from the gravid female worm. Hence, a female *D. medinensis* present in the host’s body at the time of injection becomes gravid and emerges from the host, but the larvae are not viable and fail to infect copepods and mammalian hosts. This mechanism of action for FLBZ is supported by laboratory experiments in ferrets [34]. Of note, the apparent village level effects of FLBZ in this study are likely attenuated because only half of the dogs in each village enrolled in the 2019 FLBZ clinical trial were randomized to receive a subcutaneous injection of FLBZ. Because the 2019 FLBZ trial randomized dogs in each enrolled village to receive either FLBZ or control, we are not able to separate the effects of FLBZ from any possible effects of the trial at the village level. Therefore, we are limited in our ability to say that this decrease in emerging *D. medinensis* 22 months is due to FLBZ. Because the second FLBZ trial randomized treatment of FLBZ at the village level, we hope to further explore this question when analyzing the results.

This study incorporated data provided by village supervisors and dog owners on the month when villages initiated the proactive tethering program. This initiative gave dog owners a monetary incentive to keep their dogs tethered to a heavy object to prevent them from accessing bodies of surface water. Proactive tethering is one of many behavioral interventions currently practiced in villages endemic for GWD and overseen by PNEVG-T, and these other interventions may be why we observe a decline in emerging *D. medinensis* females in dogs in villages both enrolled and not enrolled in the 2019 field trial (Fig 2A). However, we were unable to control for other behavioral interventions such as timing of temephos application due to the data being unavailable. Additionally, the 2019 study enrolled villages with high worm burdens in specific regions in Chad, while the control villages cover a wider area along the Chari River. We tried to account for differences in FLBZ-treated and control villages by including a variable for being enrolled in the 2019 FLBZ study ($\beta_{\text{1}}$), but it is possible that some uncontrolled bias between treated and control villages remains.

Tethering dogs restricts their access to shared bodies of water and, thus, reduces transmission of *D. medinensis* to other mammalian hosts that may share these water sources (i.e., humans, cats, and untethered dogs). We would expect to observe a reduction in emerging *D. medinensis* worms approximately 10–14 months after the start of tethering. This was supported by our analysis. However, proactive tethering also was hypothesized to make dogs more available for active surveillance to identify emerging *D. medinensis* females in the first generation. Our analysis did not find an immediate positive effect of proactive tethering on the number of emerging worms in the first generation, which could indicate that surveillance was already very good or that the effect of proactive tethering on surveillance intensity was delayed. A recent mathematical simulation model found that higher tethering compliance was predicted to decrease *D. medinensis* infections in dogs, compared to other interventions, over a five-year period, particularly in endemic villages located in western and central areas in Chad [42]. Our analysis found that proactive tethering resulted in a reduction in the predicted number of *D. medinensis* compared to no proactive tethering in the second-generation, as evidenced by the reduction in emerging *D, medinensis* in the third seasonal cycle, but this trend does not continue over time as evidenced by the non-significant interaction between the second-generation effect of proactive tethering and time (time since proactive tethering). This could be an indication that compliance in tethering dogs wanes over time or that improved surveillance based on the proactive tethering program was delayed. A longer observation window or field study of owner and supervisor knowledge, attitudes, and practices over time might serve to disentangle the empirically observed trend in the population.

This study has wider welfare implications for dogs. The proactive tethering program is closely monitored by PNEVG-T and overseen by trained veterinarians. With FLBZ appearing to reduce the number of emerging *D. medinensis* in dogs, proactive tethering as an intervention could be reduced. As dogs are mainly kept outdoors, they are exposed to other parasites that can cause disease. FLBZ is a potent anthelmintic which is effective in reducing numerous helminth parasites in pigs [43]. In fact, dogs treated with FLBZ in the 2021 FLBZ trial were at a lower risk for detecting cutaneous myiasis compared to control dogs [44], which suggest FLBZ may aide in controlling and preventing parasites other than *D. medinensis* and improve overall health in these dogs.

## Conclusion

As the burden of GWD in dogs remains high compared to humans and other animal hosts, it is important not only to find alternative intervention methods to interrupt the transmission of *D. medinensis*, but also to gain a better understanding of the timing and effects of interventions used individually and concurrently. Results from our analysis suggest that FLBZ may act on the developing *D. medinensis* within the host, thus reducing transmission in the second-generation of *D. medinensis*. FLBZ used concurrently with existing behavioral interventions may be an important tool in reducing the incidence of *D. medinensis* in animal hosts, although further studies are needed to separate out the effects of FLBZ from the potential effects of the clinical trial.

## Supporting information

S1 Fig Diagnostic graphs comparing the expected vs. observed fit of the residuals from Poisson and negative binomial models. (DOCX)
