# Supplementary material for: Concurrent therapeutic and behavioral interventions are associated with a reduced number of emerging Dracunculus medinensis worms in dogs in Chad
Source: PLoS Negl Trop Dis. 2026 Feb 2;20(2):e0012896. doi: 10.1371/journal.pntd.0012896 (PMC12880749; doi:10.1371/journal.pntd.0012896)

Supplemental Information

S1 Fig. Diagnostic graphs comparing the expected vs. observed fit of the residuals from Poisson and negative binomial models


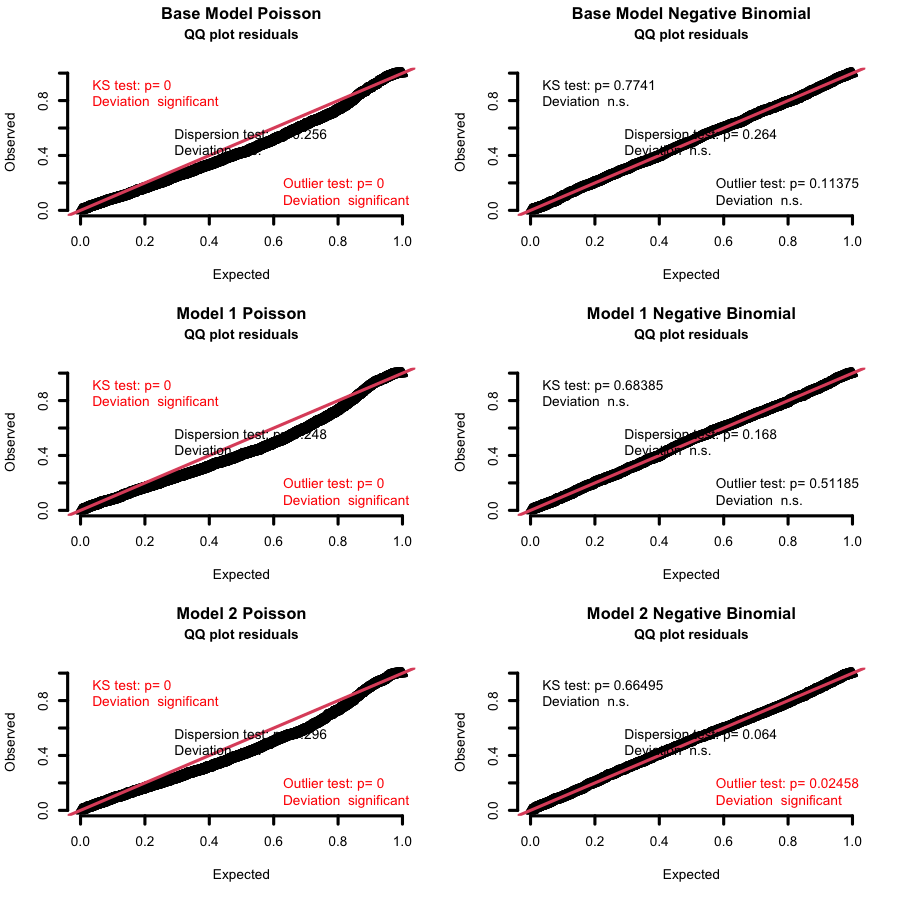


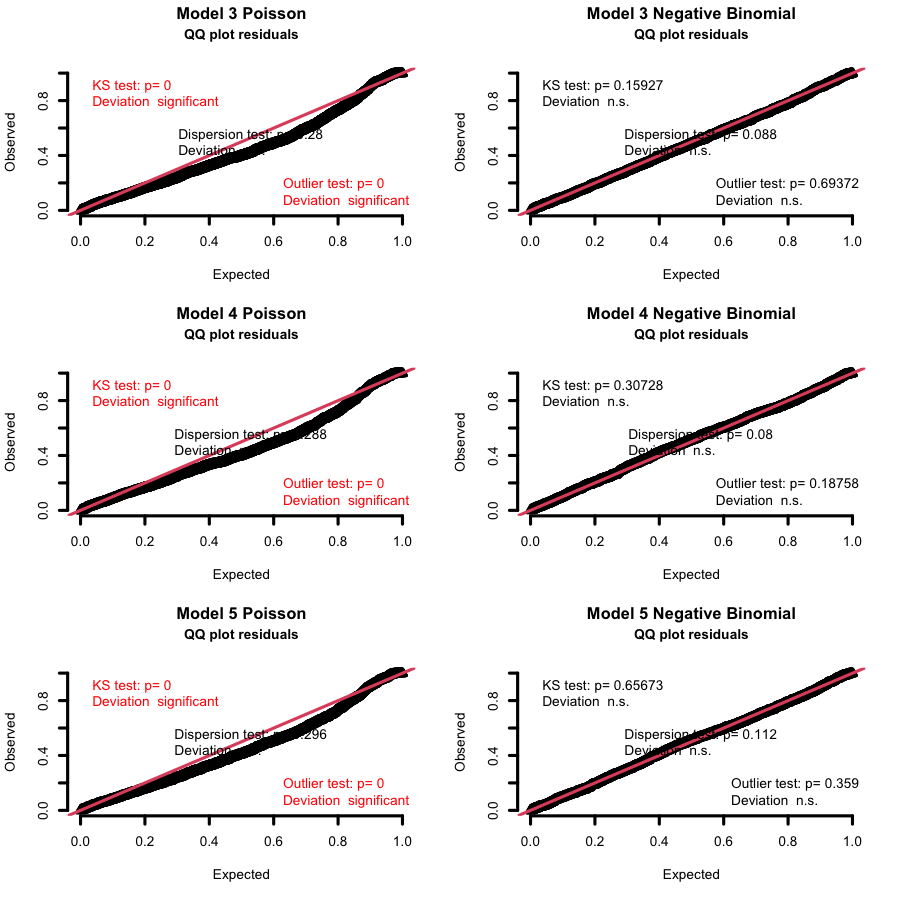


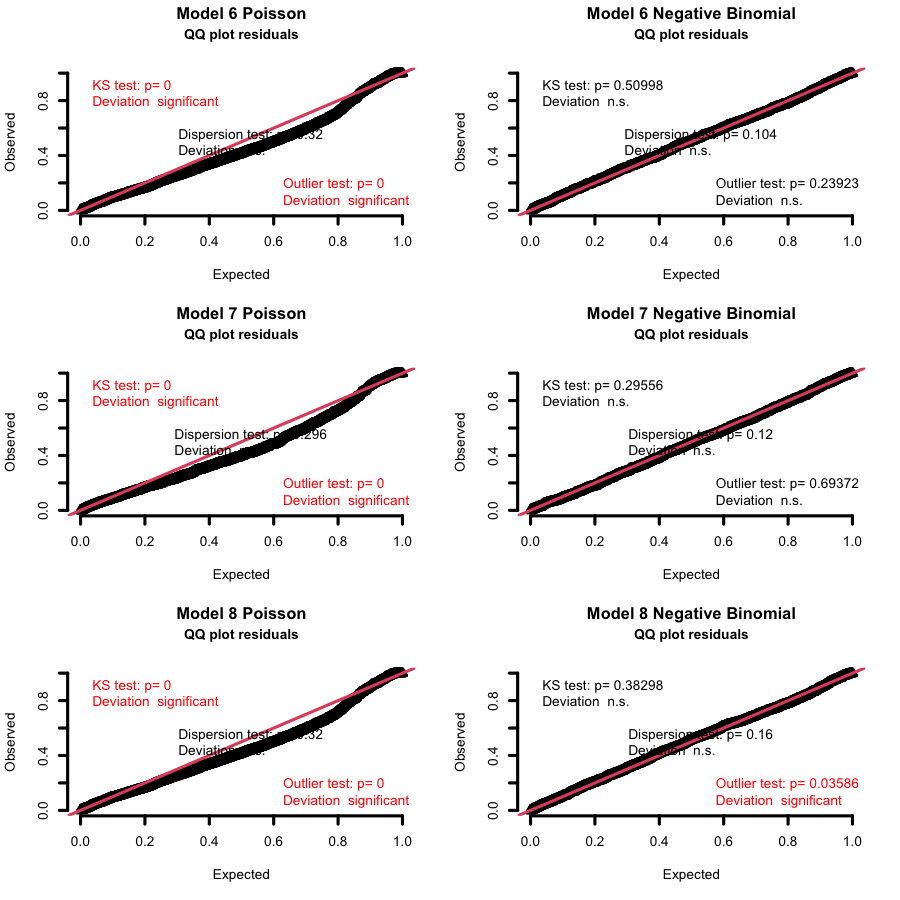


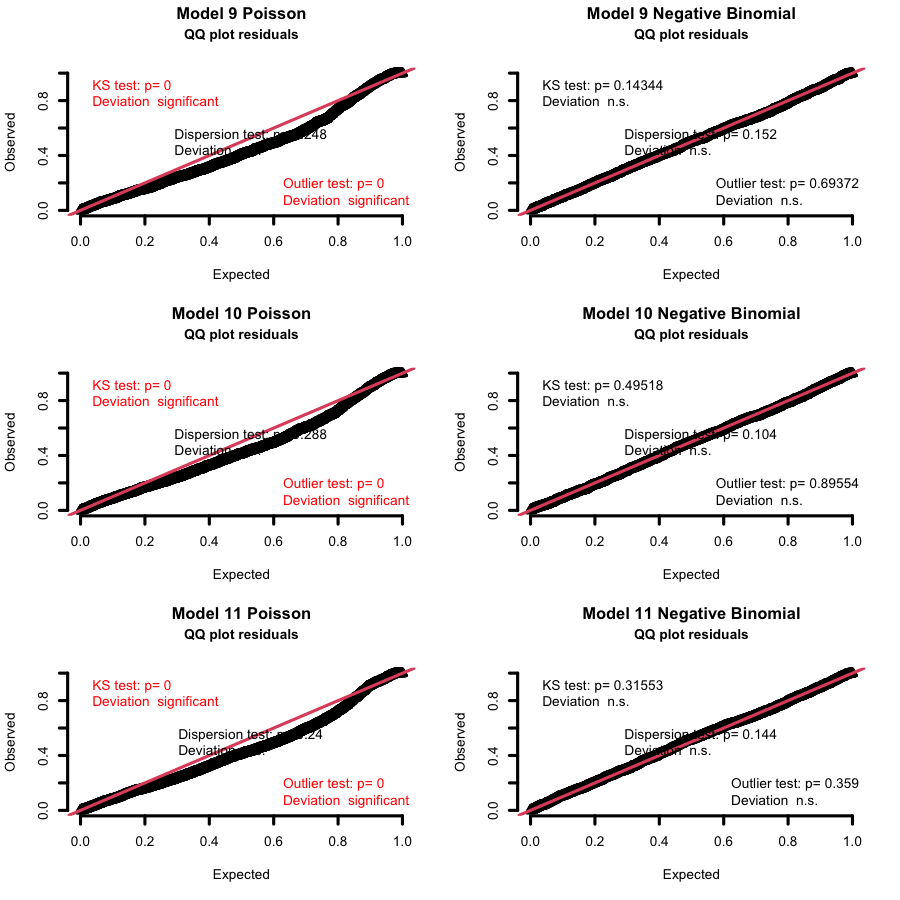


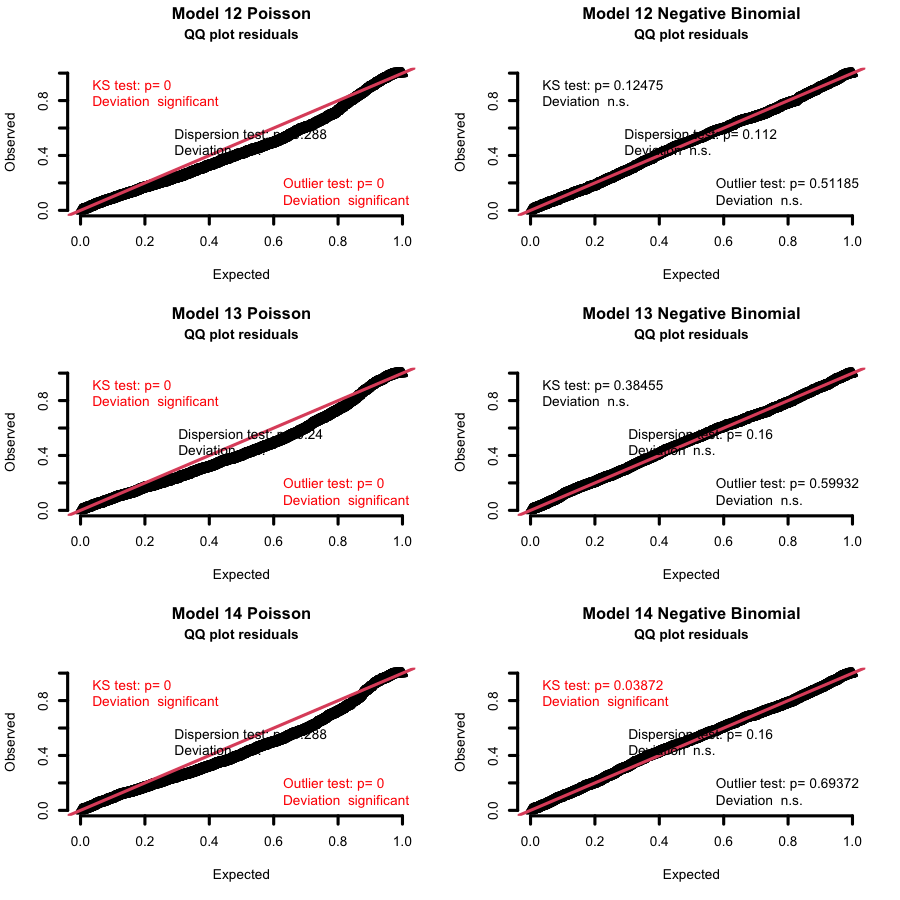


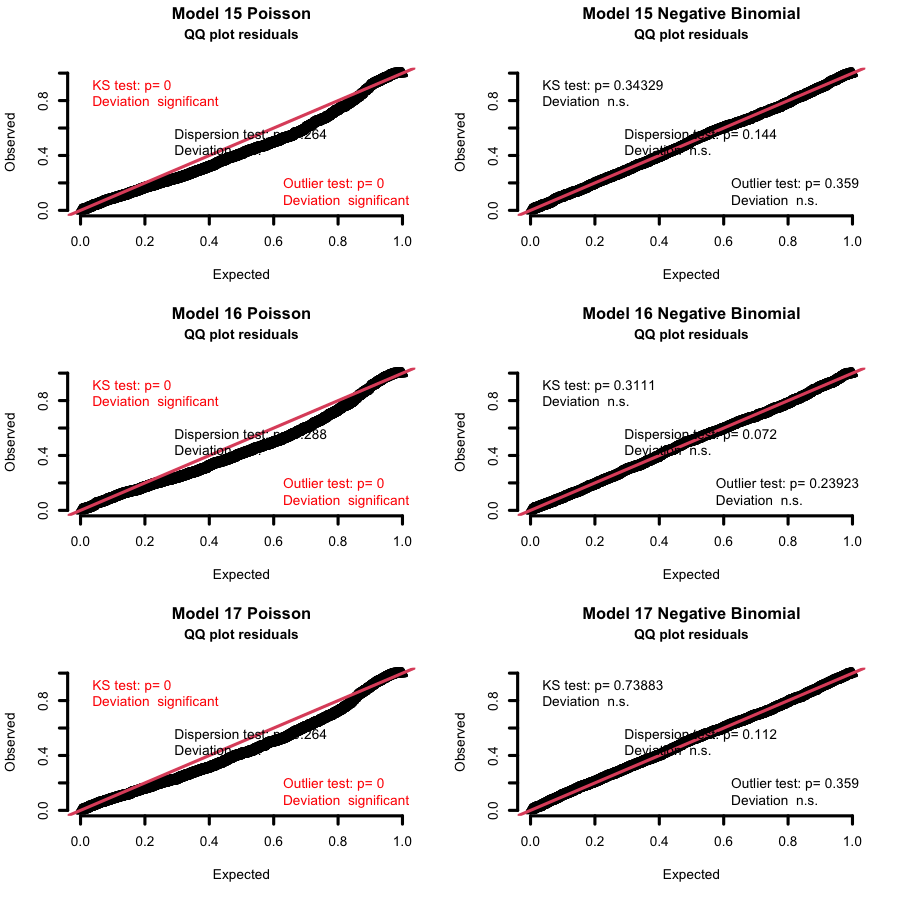


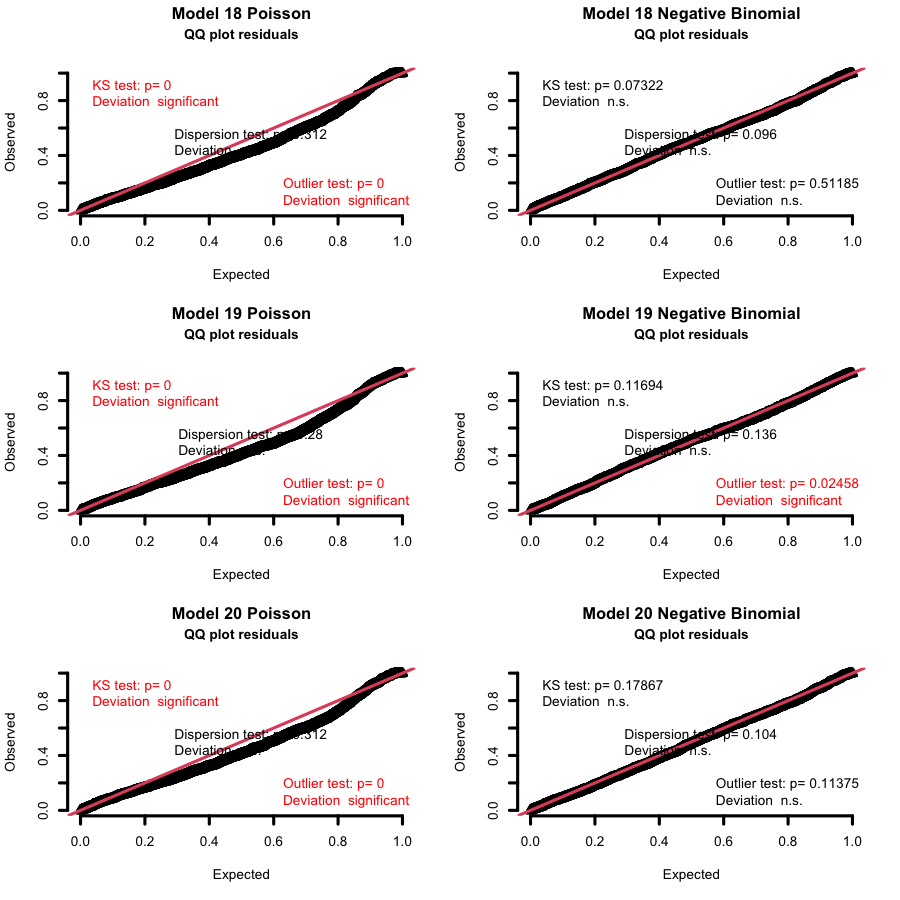


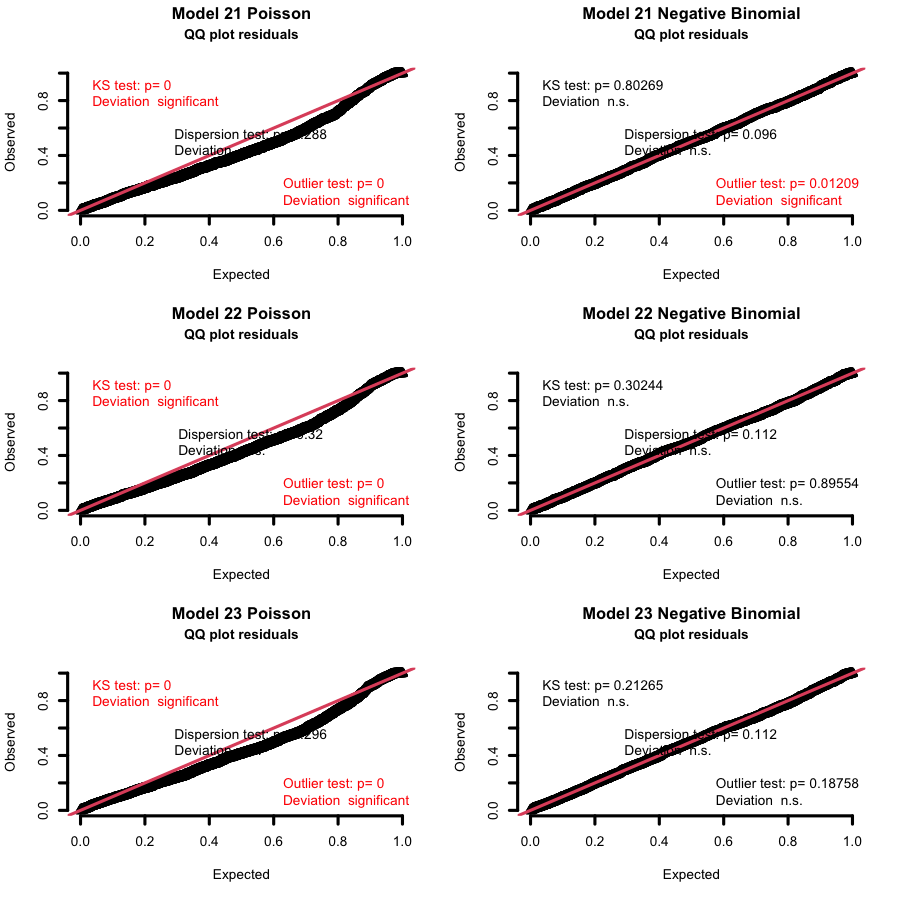


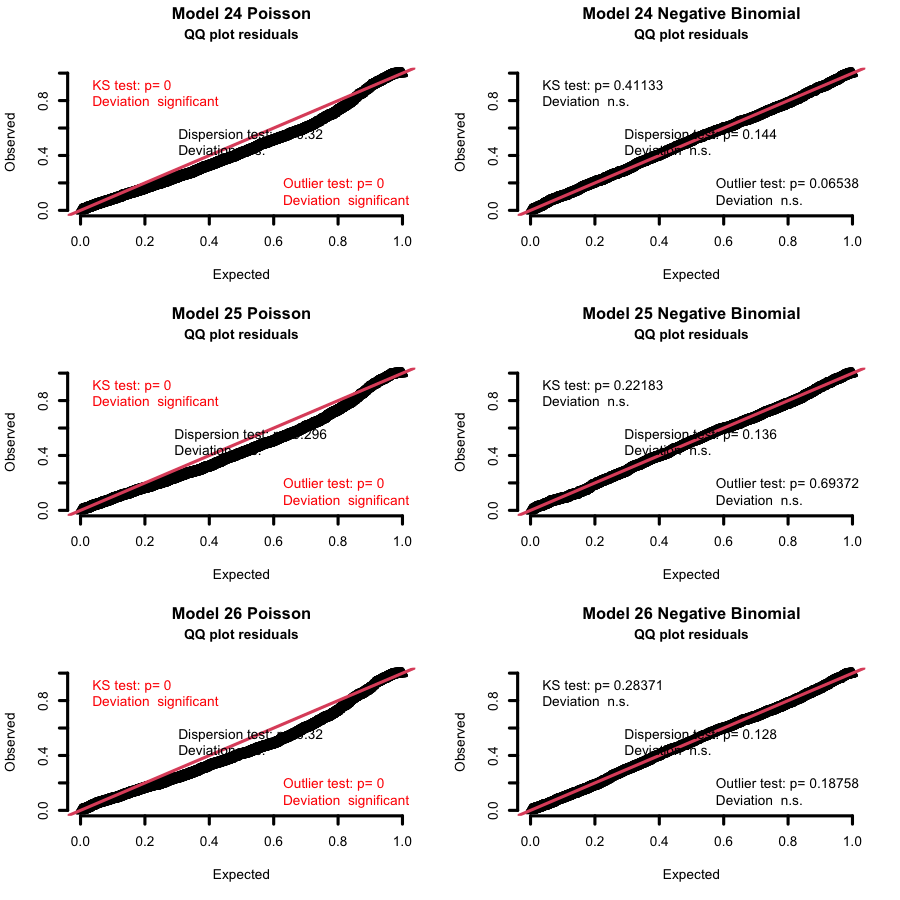


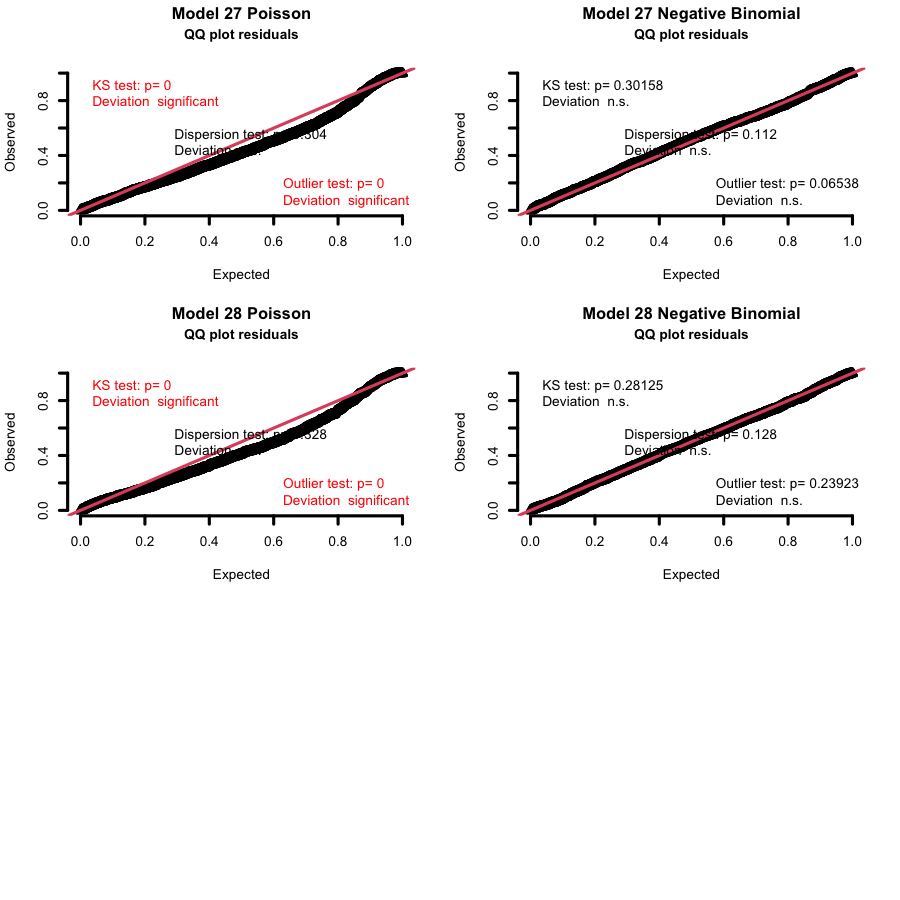

Supplement: S1 Fig — (DOCX) [file pntd.0012896.s001.docx]
